# miR-215 Modulates Ubiquitination to Impair Inflammasome Activation and Autophagy During *Salmonella* Typhimurium Infection in Porcine Intestinal Cells

**DOI:** 10.3390/ani15030431

**Published:** 2025-02-04

**Authors:** Carmen Entrenas-García, José M. Suárez-Cárdenas, Raúl Fernández-Rodríguez, Rocío Bautista, M. Gonzalo Claros, Juan J. Garrido, Sara Zaldívar-López

**Affiliations:** 1 Immunogenomics and Molecular Pathogenesis Group, UIC Zoonosis and Emergent Diseases ENZOEM, Department of Genetics, University of Cordoba, 14014 Cordoba, Spain; b82engac@uco.es (C.E.-G.); b52sucaj@uco.es (J.M.S.-C.); b42feror@uco.es (R.F.-R.); 2 GA-14 Research Group, Maimónides Biomedical Research Institute of Córdoba (IMIBIC), 14004 Cordoba, Spain; 3 Plataforma Andaluza de Bioinformática, Supercomputing and Bioinnovation Center (SCBI), Universidad de Málaga, 29590 Malaga, Spain; rociobm@uma.es (R.B.); claros@uma.es (M.G.C.); 4 Institute of Biomedical Research in Malaga (IBIMA), IBIMA-RARE, 29590 Malaga, Spain; 5 Institute for Mediterranean and Subtropical Horticulture “La Mayora” (IHSM-UMA-CSIC), 29590 Malaga, Spain; 6 Department of Molecular Biology and Biochemistry, Universidad de Málaga, 29010 Malaga, Spain; 7 CIBER de Enfermedades Raras (CIBERER) U741, 29071 Malaga, Spain

**Keywords:** microRNA, salmonellosis, inflammation, zoonosis, immune response

## Abstract

The host response to *S.* Typhimurium infection can be post-transcriptionally regulated by miRNAs. In this study, we investigated the role of miR-215 using both in vivo porcine infection models and in vitro intestinal epithelial cell lines. Several miRNAs were found to be dysregulated in the porcine ileum during infection with wild-type and SPI2-defective mutant strains of *S.* Typhimurium, with some changes being SPI2-dependent. Notably, miR-215 was significantly downregulated during infection. To explore its functional role, gain-of-function experiments were performed by transfecting porcine intestinal epithelial cells (IPEC-J2) with a miR-215-5p mimic, followed by label-free quantitative (LFQ) proteomic analysis. This analysis identified 157 proteins, of which 35 were downregulated in response to miR-215 overexpression, suggesting they are potential targets of this miRNA. Among these, E2 small ubiquitin-like modifier (SUMO)-conjugating enzyme UBC9 and E3 ubiquitin-ligase HUWE1 were identified as key targets, both of which are upregulated during *S*. Typhimurium infection. The miR-215-mediated downregulation of these proteins resulted in a significant decrease in overall ubiquitination, a process crucial for regulating inflammasome activation and autophagy. Consistently, inflammasome markers caspase 1 (CASP1) and apoptosis-associated speck-like protein containing a CARD (ASC), as well as autophagy markers microtubule-associated protein 1A/1B-light chain 3 (LC3B) and Ras-related protein Rab-11 (RAB11A), showed decreased expression in miR-215 mimic-transfected and infected IPEC-J2 cells. To further validate these findings, human intestinal epithelial cells (HT29) were used as a complementary model, providing additional insights into conserved immune pathways and extending the observations made in the porcine system. Overall, our findings demonstrate that miR-215 plays a significant role in modulating host inflammasome activation and autophagy by targeting proteins involved in ubiquitination during *S.* Typhimurium infection.

## Simple Summary

In this study, we investigated the role of microRNA-215 (miR-215) during *Salmonella* Typhimurium (*S*. Typhimurium) infection using a combination of in vivo porcine models and in vitro epithelial cell lines. Several dysregulated microRNAs (miRNAs) were identified in the infected porcine ileum, with miR-215 showing significant downregulation. Gain-of-function experiments in porcine (IPEC-J2) and human (HT29) intestinal epithelial cells revealed that miR-215 overexpression inhibits ubiquitination, subsequently downregulating key host immune response pathways such as autophagy and inflammasome activation. Our findings demonstrate that miR-215, downregulated during *Salmonella* Typhimurium infection in porcine ileum, plays a crucial role in regulating the host immune response by targeting proteins involved in ubiquitination, thereby influencing inflammasome activation and autophagy.

## 1. Introduction

Salmonellosis is one of the leading foodborne zoonoses worldwide, ranking second within the European Union (EU) and affecting over 60,000 people every year [1]. With the establishment of control programs in poultry, swine have emerged as an important source of infection, along with turkey and cattle. Pigs remain as carriers after infection, not showing any signs of disease and thus silently spreading bacteria through the food chain, which represents a considerable risk to public health. Given the importance of the pork industry in the economy of many producing and exporting countries, economic losses caused by a widespread disease such as salmonellosis can be very significant. Among all disease-causing serovars, one of the most frequently isolated serovars in pigs in Europe is *Salmonella enterica* serovar Typhimurium (hereinafter *S*. Typhimurium) [1,2].

The interaction between bacterial pathogens and the host immune system is a complex process involving numerous molecular mechanisms. In this context, the regulation of inflammasome activation and autophagy plays a pivotal role in the innate immune response to bacterial infections. MicroRNAs have emerged as key regulators of the infection process, modulating gene expression at the post-transcriptional level [3,4,5]. Mature miRNAs have the capacity to bind to hundreds of target genes by complementarity with the mRNA, typically in the 3′ untranslated region (UTR). This binding results in the silencing of the target mRNA through mechanisms such as mRNA degradation or inhibition of its translation by ribosomes [6]. It has been demonstrated that infections by bacterial pathogens can be modulated by miRNAs in a very complex manner, altering not only immune pathways, but also cell death, cytoskeletal organization and autophagy [7,8]. It has been demonstrated that miRNAs such as miR-194, miR-155, miR-125, let-7i and miR-21 can regulate host responses to *S*. Typhimurium infection [9,10]. For instance, miR-194 has been shown to modulate inflammatory response by regulating the toll-like receptor 4 (TLR4)-mediated signaling pathway [11]; miR-21 induces macrophage activation [12]; and let-7i-3p is involved in bacterial adhesion and intracellular replication [9]. More recently, it has been shown that alteration of the intestinal mucosal miRNA profile (miRNome) during *S*. Typhimurium infection contributes positively to the spread of infection, mainly as a consequence of the downregulation of miR-15a, miR-15b, miR-16, miR-22, miR-421, miR-744 and let-7i [13].

To gain deeper insight into the regulatory process exerted by miRNAs, it is crucial to understand the molecular pathogenesis of *Salmonella* infection. After traveling through the gastrointestinal tract, *S*. Typhimurium invades the intestinal mucosal epithelium, causing a strong inflammatory reaction [14,15]. *S*. Typhimurium employs effector proteins, encoded by pathogenicity islands 1 and 2 of the bacterial chromosome (SPI1 and SPI2, respectively) [16]. These proteins are secreted by the bacterial type III secretion system and allow entry into epithelial mucosa enterocytes, forming the *Salmonella*-containing vacuole and thereby creating a suitable environment for intracellular permanence and replication [17]. Bacterial entry into cells triggers the innate immune response, characterized by synthesis of pro-inflammatory cytokines, which further promote the recruitment of innate immunity cells (e.g., macrophages, neutrophils) and magnify the inflammatory response. *Salmonella* invasion is recognized by the nucleotide oligomerization domain producing leucine rich repeat-like receptors (NLR), such as the NLR family caspase recruitment (CARD) domain-containing protein 4 (NLRC4) and the NLR family pyrin domain-containing 3 (NLRP3), which activate the inflammasome complex. Inflammasome is composed of an NLR, an adaptor protein (ASC), and an effector protein (CASP1), and leads to the production of interleukin 1β (IL1β) and interleukin 18 (IL18) [18]. Elimination of the pathogen and unwanted proteins is carried out by autophagy, which is a cellular degradation and recycling process that also contributes to the regulation of inflammation.

Ubiquitination, a post-translational modification involving the covalent attachment of ubiquitin molecules to target proteins, is a key regulatory mechanism for both inflammasome activation and autophagy [19]. The conjugation of ubiquitin is mediated by E1 ubiquitin-activating enzymes, E2 ubiquitin-conjugating enzymes, and E3 ubiquitin ligases. Upon infection, ubiquitin-coated proteins or *Salmonella* are recognized by autophagy receptors and sent to autophagosomes for degradation, which will fuse with lysosomes to form the autolysosome [19,20]. Also, the ubiquitination/deubiquitination of inflammasome components such as ASC, NLRP3 or CASP1 regulates the activation of the inflammasome, which helps maintaining inflammatory homeostasis [21]. *Salmonella* has developed mechanisms to counteract host defense by hijacking host ubiquitin pathways, thus evading the immune response [19]. Some *Salmonella* SPI1 and SPI2 effectors have E3 ligase activity (i.e., SopA, SspH1, SspH2 and SlrP) [22,23,24]. SopA can stimulate host E3 ubiquitin ligases, resulting in their proteasomal degradation during infection, SspH1 inhibits nuclear factor kappa-light-chain-enhancer of activated B cells (NFκB) pathway by manipulating host ubiquitination, and SspH2 enhances interleukin 8 secretion [19]. On the contrary, other virulence factors such as SseL have deubiquitinase (DUB) activity [25].

As previous studies have demonstrated that expression of miRNAs changes during *S*. Typhimurium infection in porcine ileum [11], here, we aimed to evaluate if a *S*. Typhimurium strain lacking SPI2, which is essential for intracellular replication and immune response activation, induces miRNA dysregulation during infection. We identified several miRNAs that are differentially expressed, choosing downregulated miR-215 for further functional analysis in intestinal cell lines. MiR-215 is a highly conserved microRNA implicated in various biological processes, including cell cycle regulation, differentiation, and stress responses [26,27], with emerging evidence suggesting a potential role in modulating immune responses and inflammation [28,29]. However, its precise function in regulating ubiquitination and its effects on inflammasome activation and autophagy during *S.* Typhimurium infection in porcine intestinal cells remain poorly understood. We hypothesize that miR-215 influences the expression of genes involved in the ubiquitination machinery, thereby impacting inflammasome activation and the induction of autophagy in response to *S.* Typhimurium infection.

This study aims to uncover the molecular mechanisms underlying innate immune responses in porcine intestinal cells during *S.* Typhimurium infection, offering potential insights for developing innovative therapeutic strategies to combat porcine salmonellosis and enhance food safety.

## 2. Materials and Methods

### 2.1. Experimental Infection and Tissue Sample Collection

Twelve recently weaned piglets (commercial hybrids of Landrace × Large White × Pietrain) of approximately 4 weeks of age were used in this study. Experimental procedures were performed at University of León (Spain) following a methodology previously described [14]. Briefly, piglets were divided into the following three groups: four pigs were orally inoculated with 5 mL of saline solution (control group, C); four pigs were orally inoculated with 5 mL of culture broth (brain heart infusion, BHI, Sigma-Aldrich, St. Louis, MO, USA) containing 10^8^ colony forming units (CFU)/mL of the *S*. Typhimurium strain SL1344 (ST group); and the remaining four pigs were inoculated with 5 mL of culture broth (BHI, Sigma-Aldrich, St. Louis, MO, USA) containing 10^8^ CFU/mL of a SL1344 SPI2-defective mutant (MUT group) kindly provided by Dr. Carmen Aguilar (University Würzburg, Germany). Prior to in vivo infection and following a previously published methodology [30], bacterial invasion was assessed in both strains, confirming comparable invasion capacities. All animals in the ST and MUT groups tested positive for *Salmonella* in fecal samples by microbiological cultures, while all animals from the C group tested negative. At 2 days post-infection (dpi), animals were humanely euthanized, and ileum sections were collected and snap-frozen in liquid nitrogen for further processing.

### 2.2. RNA Isolation

RNA was isolated from ileal mucosa, as previously described [11]. Briefly, intestinal mucosa was scraped from the intestinal luminal surface with a sterile razor blade, and immediately homogenized in mirVana miRNA isolation kit lysis buffer (Ambion Inc., Austin, TX, USA) using a rotor–stator homogenizer. RNA extraction was performed following manufacturer instructions. Eluted RNA was treated with TURBO DNA-free™ Kit (Ambion Inc., Austin, TX, USA) and RNA integrity was assessed in the Agilent Bioanalyzer 2100 (Agilent Technologies, Palo Alto, CA, USA); only samples with RNA integrity numbers (RIN) ≥ 8 were used for sequencing.

### 2.3. Small RNA Sequencing

Library preparation and small RNA sequencing were performed at the Genomics core service at the Institute of Biomedical Research Barcelona (IRB), and data obtained were bioinformatically pre-processed and analyzed at the Supercomputing and Bioinnovation Center of the University of Malaga (UMA-SCBI), following a methodology previously described [11]. Data were graphed with Volcanoser [31], miRNA sequences were retrieved from miRbase and aligned using Clustal Omega [32], and structure was predicted using mfold [33].

### 2.4. Cell Culture

IPEC-J2 cells, a non-transformed epithelial cell line derived from the jejunum of a neonatal piglet, were used as the primary model for our experiments due to their porcine origin, which aligns with our in vivo infection model. These cells were cultured in Dulbecco’s modified Eagle’s and Ham’s F-12 medium (Capricorn Scientific GmbH, Ebsdorfergrund, Germany) supplemented with 5% fetal bovine serum (Gibco; Thermo Fisher Scientific, Inc., Waltham, MA, USA), in a humidified atmosphere with 5% CO_2_ at 37 °C. To evaluate the invasion capacities of both *Salmonella* strains (ST and MUT) used in the in vitro experiments, gentamicin protection assays and quantification using *S*. Typhimurium-specific probes and primers were performed as previously reported [30], which confirmed their ability to invade the cells with no statistical differences between them. For specific functional validation experiments requiring protein-level analysis, significant challenges due to the limited availability and poor performance of porcine-specific antibodies were encountered. To overcome this technical limitation and ensure robust protein detection, the study was supplemented with the human epithelial HT29-MTX-E12 cell line (hereinafter HT29). This well-characterized human cell line allows for more reliable protein quantification using widely available human-specific antibodies. HT29 cells were cultured in Dulbecco’s modified Eagle’s medium high glucose (Biowest) supplemented with 10% fetal bovine serum and 1% non-essential amino acids (both from Gibco; Thermo Fisher Scientific, Inc., Waltham, MA, USA). By using both cell lines, it was possible to leverage the species-specific advantages of IPEC-J2 cells while addressing the technical constraints in protein analysis using HT29 cells.

### 2.5. Transfection and Infection

microRNA mimic hsa-miR-215-5p (C-300570-05, homologous to ssc-miR-215), and mimic negative control (CN-001000-01-05) were obtained from miRIDIAN (Dharmacon Inc., Lafayette, CO, USA). Mimic miR-215 and negative control were transfected using Lipofectamine™ RNAiMAX (Thermo Scientific Inc., Waltham, MA, USA), following the direct transfection protocol recommended by the manufacturer, at a final miRNA concentration of 50 nM. Briefly, 7.5 × 10^4^ or 2.5 × 10^5^ cells (per well) were seeded, 24 h before transfection, on 24- or 6-well plates, respectively. Following the objectives of the experiment, 24-well plates were used for RNA isolation and 6-well plates for protein isolation. Transfected cells were incubated at 37 °C in a 5% CO_2_ humidified atmosphere for 48 h, and then infected with *S*. Typhimurium phage type DT104 [11] (optical density at 600 nm (OD_600_) = 0.8, multiplicity of infection (MOI) = 100). After 1 h of infection, the medium was replaced with fresh medium containing gentamicin (50 µg/mL) to kill extracellular bacteria and incubated for 2 h and 24 h. At that time, cells were scraped and collected using radio-immunoprecipitation assay (RIPA) buffer. The transfection of miRNA was confirmed using quantitative polymerase chain reaction (qPCR) analysis, as previously described [11].

### 2.6. Protein Sample Preparation and Liquid Chromatography with Tandem Mass Spectrometry (LC-MS/MS) Proteomics

Protein concentration was measured using the Pierce™ BCA Protein Assay Kits (Thermo Scientific Inc., Waltham, MA, USA), and 100 µg were sent to the Proteomics Facility at Research Support Central Service, University of Cordoba (SCAI-UCO), for sample preparation and analysis. Protein extracts underwent clean-up in one dimensional sodium dodecyl sulfate-polyacrylamide gel electrophoresis with 10% polyacrylamide. After electrophoresis at 100 V, gel was stained with Coomassie Blue and protein bands were cut out and stored in water until digestion. Protein bands were first destained in 200 mM ammonium bicarbonate (AB)/50% acetonitrile, then in 100% acetonitrile, reduced with 20 mM dithiothreitol and incubated in 25 mM AB at 55 °C. After cooling to room temperature, free thiols were alkylated with 40 mM iodoacetamide. Following two AB washes, proteins were digested with trypsin (Promega, Madison, WI, USA) and incubated overnight at 37 °C. Digestion was stopped by adding trifluoroacetic acid to a final concentration of 1%. Digested samples were dried and resuspended in 0.1% formic acid (FA).

Nano liquid chromatography (LC) was performed on a Dionex Ultimate 3000 nano ultra performance LC (Thermo Scientific Inc., Waltham, MA, USA) with a C18 75 µm × 50 Acclaim Pepmap column (Thermo Scientific Inc., Waltham, MA, USA). Peptides were loaded onto a 300 µm × 5 mm Acclaim Pepmap precolumn (Thermo Scientific Inc., Waltham, MA, USA) in 2% acetonitrile/0.05% trifluoroacetic acid, then separated at 40 °C with a gradient of buffer A (water with 0.1% FA) and buffer B (20% acetonitrile with 0.1% FA) over 85 min of chromatography. Peptide cations were ionized and analyzed on a Thermo Orbitrap Fusion (Q-OT-qIT, Thermo Scientific Inc., Waltham, MA, USA), operated in positive mode with survey scans at 120K resolution. Tandem mass spectrometry (MS/MS) involved quadrupole isolation, collision-induced dissociation fragmentation, and rapid scan MS analysis, sampling only precursors with charge states 2–5. The instrument operated in top speed mode with 3 s MS2 cycles, excluding precursors dynamically.

MS2 spectra were analyzed using MaxQuant software v. 1.6.17.0 [34], with the Andromeda search engine set against a Uniprot_proteome_Sus-scrofa_Oct21 database. Peptides were generated through tryptic digestion with up to one missed cleavage, fixed carbamidomethylation of cysteines, and variable oxidation of methionine. The mass tolerance was 10 ppm, and product ions were searched with 0.6 Da tolerance. Peptide matches were filtered to a 1% false discovery rate (FDR). Quantification was conducted using the MaxLFQ label-free method [35], using retention time alignment and match-between-runs protocol. Heatmaps were generated using the Morpheus software (https://software.broadinstitute.org/morpheus/, accessed on 12 July 2024).

### 2.7. Western Blotting and Label-Free Quantitation

Briefly, 30 μg of protein were loaded per lane, separated on 12% SDS-PAGE gels, and blotted onto polyvinylidene difluoride membranes (Immobilon-P, Merck Millipore Inc., Burlington, MA, USA). Membranes were blocked in 5% non-fat milk for 1 h at room temperature, followed by overnight incubation at 4 °C with the following primary antibodies: Glyceraldehyde 3-phosphate dehydrogenase (GAPDH) (G8795; 1:10,000 dilution; Merck Millipore Inc., Burlington, MA, USA); LC3B (#3868; 1:1,000 dilution; Cell Signaling Technology Inc., Danvers, MA, USA); CASP1 (sc-398715; 1:1,000 dilution; Santa Cruz Biotechnology Inc., Dallas, TX, USA); ASC (sc-514414; 1:1,000 dilution; Santa Cruz Biotechnology Inc., Dallas, TX, USA); ubiquitin (sc-271289; 1:5,000 dilution; Santa Cruz Biotechnology Inc., Dallas, TX, USA); IL1β (sc-12742; 1:1,000 dilution, Santa Cruz Biotechnology Inc., Dallas, TX, USA); RAB11A (sc-166912; 1:1,000 dilution; Santa Cruz Biotechnology Inc., Dallas, TX, USA); and caspase 11 (CASP11) (sc-374615; 1:1,000 dilution; Santa Cruz Biotechnology Inc., Dallas, TX, USA). Membranes were further incubated with the secondary antibody Anti-Mouse immunoglobulin G (whole molecule)–peroxidase antibody produced in rabbit (A9044; 1:10,000; Merck Millipore Inc., Burlington, MA, USA) at room temperature during 1h, and immunoblots were visualized using an Immobilon UltraPlus Western HRP Substrate chemiluminescence kit (Merck Millipore Inc., Burlington, MA, USA) in a Chemidoc MP imaging system. The relative protein expression was analyzed using ImageJ software 1.4 (National Institutes of Health), using GAPDH as a control.

### 2.8. Reverse Transcription-Quantitative Real-Time PCR (RT-qPCR) and Statistical Analysis

Total RNA from cell cultures was extracted with TRIzol reagent (Thermo Scientific Inc., Waltham, MA, USA) as described in the manufacturer’s protocol. For the miRNA expression analysis, 100 ng of total RNA per sample was reverse transcribed to cDNA as previously reported [36]; for mRNA analysis, 500 ng of RNA was reverse transcribed using qScript™ cDNA synthesis kit (QuantaBio LLC., Beverly, MA, USA), following the manufacturer’s instructions. PCR reaction mixes and settings were as previously described [11], and primer sets are included in Table S3. Differential gene expression was calculated using the 2^−ΔΔCt^ method [37] using the GenEx software (GenEx 6, bioMCC, Freising, Germany), and statistical analysis and graphs were performed using GraphPad Prism software (v9.4.1). Comparison between groups was performed using unpaired *t*-test or analysis of variance with Tukey’s multiple comparison’s test, setting *p* = 0.05 as threshold of significance.

## 3. Results

### 3.1. miRNA Profiling and Characterization of miR-215 During S. Typhimurium Infection in Porcine Ileum

To investigate the role of miRNAs during *S*. Typhimurium (ST) infection, we per-formed small RNA sequencing on ileal samples from pigs infected with wild-type ST, a SPI2-defective mutant (MUT), and uninfected controls (C). Our analysis identified a total of 334 known porcine miRNAs across all samples (Table 1 and Table S1). Comparison between infected and control samples revealed differences in miRNA expression patterns, with 43 miRNAs differentially expressed in ST-infected samples and 27 in MUT-infected samples (*p* value < 0.05). It was observed that some miRNAs behaved similarly in both groups ST and MUT, such as upregulated miR-29b, miR-144-5p/3p, miR-181b-3p, miR-301b-3p, miR-374b-3p and miR-20b, as well as downregulated miR-31-5p, miR-676-3p, miR-574-5p and miR-9.

Notably, we observed that the modulation of several miRNAs was influenced by the presence of a functional SPI2. Among these, miR-215 showed significant downregulation in ST-infected samples compared to the C group. No significant changes were observed in MUT-infected samples, suggesting that the downregulation of miR-215 during ST infection may be associated with SPI2 function, potentially through direct or indirect mechanisms.

Bioinformatic analysis revealed that miR-215 is highly conserved across species, including humans, pigs, and other mammals. Notably, only miR-215-5p has been reported in pigs, exhibiting complete sequence homology with its human counterpart. However, the sequencing of small RNAs carried out in the present work and subsequent mapping into the human database allowed us to detect that miR-215-3p is also present in swine, although it is expressed in the *S*. Typhimurium-infected ileum to a significantly lesser extent than miR-215-5p (Figure S1, Table S1). Consequently, we used the latter to investigate the function of miR-215 during *S*. Typhimurium infection using a mimic-based gain-of-function approach.

### 3.2. miR-215 Modulates Ubiquitination, Inflammasome Activation and Autophagy

**Figure 1:**
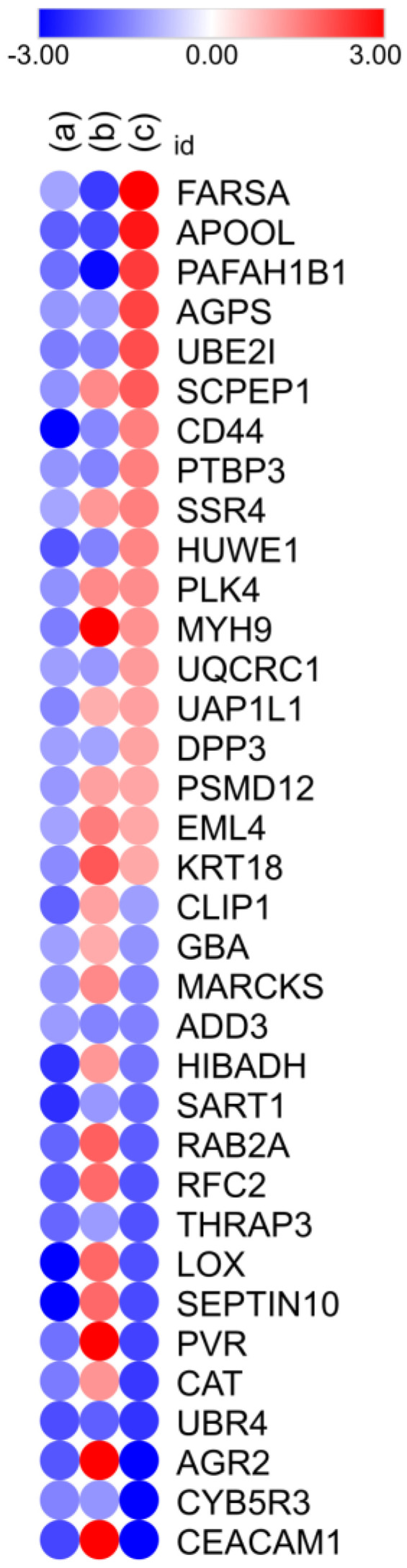
Fold change protein expression on IPEC-J2 cells in the following three experimental situations (columns) studied: (**a**) miR-215 mimic-transfected cells compared to mock control cells; (**b**) cells transfected with the miR-215 mimic and infected with *S.* Typhimurium compared to mock control cells infected with *S.* Typhimurium (infection duration 2 h); and (**c**) mock control cells infected with *S.* Typhimurium compared to mock control cells (infection duration 2 h). Red indicates overexpression and blue indicates downregulation.

To investigate the regulatory role of miR-215, porcine intestinal epithelial cells (IPEC-J2) were transfected with a miR-215-5p mimic, subsequently infected with *S.* Typhimurium, and analyzed using label-free quantitative (LFQ) proteomics. Four experimental groups were established: mock control cells (transfected with a mimic negative control), mock control cells infected with *S.* Typhimurium, cells transfected with the miR-215 mimic, and cells transfected with the miR-215 mimic and infected with *S.* Typhimurium. Transfection resulted in a 15-fold overexpression of miR-215 compared to negative controls. The primary focus of the analysis was on the regulatory capacities of the miR-215 mimic under condition (a), where miR-215 mimic-transfected cells were compared to mock control cells. This comparison aimed to identify proteins directly downregulated by the mimic, as these are potential post-transcriptional targets of miR-215. Additional comparisons explored the role of miR-215 in the context of bacterial infection: cells transfected with the miR-215 mimic and infected with *S.* Typhimurium were compared to mock control cells infected with *S.* Typhimurium (b) to study the combined effects of miR-215 overexpression and bacterial infection, while mock control cells infected with *S.* Typhimurium were compared to mock control cells (c) to examine proteomic changes induced by *S.* Typhimurium infection alone. In total, LFQ proteomics identified 162 proteins/peptides across these conditions, of which 127 were upregulated and 35 were downregulated following miR-215 mimic transfection. Proteins downregulated in condition (a) were prioritized for further analysis as potential direct targets of miR-215-5p, while the additional conditions provided a deeper understanding of how miR-215-5p modulates host responses during bacterial infection (Figure 1, Table S2).

**Figure 2:**
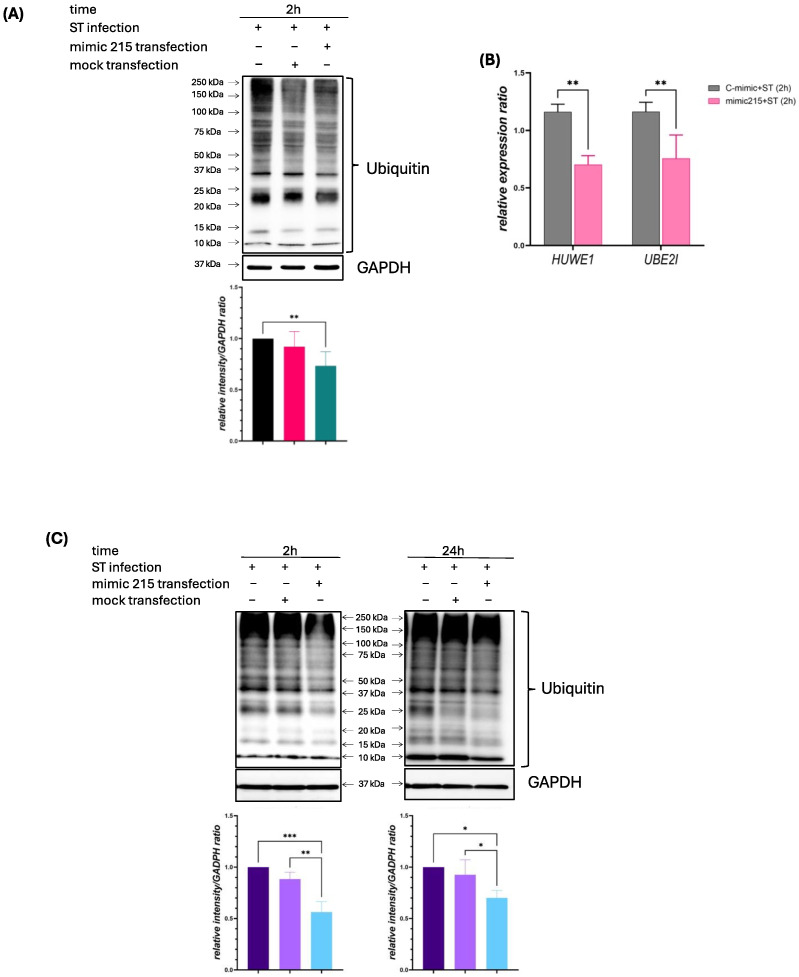
(**A**) Ubiquitin expression in IPEC-J2 cells, IPEC-J2 cells transfected with negative controls (mock) and IPEC-J2 cells transfected with miR-215 mimic, 2 h after infection with ST (ratio of the relative quantification intensity to GAPDH below each lane). (**B**) Relative gene expression (to cyclophilin A) of HUWE1 and UBE2I genes. (**C**) Ubiquitin expression in HT29 cells, HT29 cells transfected with negative controls (mock) and HT29 cells transfected with miR-215 mimic, 2 h and 24 h after infection with ST (ratio of the relative quantification intensity to GAPDH below each lane). * *p* < 0.05; **: *p* < 0.01; ***: *p* < 0.001.

Proteins that were downregulated in condition (a) and upregulated in condition (c) are likely to be regulated by miR-215-5p. This is because miR-215 was overexpressed in condition (a) due to mimic transfection, leading to the repression of its targets, while in condition (c), miR-215 was endogenously downregulated as a result of *S.* Typhimurium infection, allowing the expression of those same targets to increase. These patterns suggest a direct regulatory relationship between miR-215 and these proteins. Among these proteins, UBE2I (ubiquitin conjugating enzyme E2) and HUWE1 (E3 ubiquitin ligase) play central roles in the ubiquitination pathway. The modulation of these proteins suggests that miR-215 plays a critical role in host–pathogen interactions, particularly in processes such as inflammasome activation and autophagy. To evaluate the overall ubiquitination status and its regulation by miR-215, ubiquitin expression was analyzed in miR-215 mimic-transfected IPEC-J2 intestinal cells 2 h after infection with *S.* Typhimurium, using a human antibody capable of detecting mono- and polyubiquitinated proteins. Quantification of sample lanes, normalized to the internal control GAPDH, revealed a significant decrease (*p* < 0.01) in overall ubiquitination in miR-215 mimic-overexpressing cells infected with *S*. Typhimurium, compared to *S*. Typhimurium-infected IPEC-J2 cells without mimic transfection (Figure 2A). Additionally, HUWE1 and UBE2I gene expression levels were evaluated during infection, showing a decrease in expression when miR-215 mimic was overexpressed (Figure 2B). Although IPEC-J2 cells were chosen as the primary model due to their porcine origin, it was observed that human intestinal epithelial cells (HT29), with more physiologically relevant characteristics in their mucus layer formation, exhibit higher efficiency of *Salmonella* infection and a more robust immune response compared to other cell lines [38]. Given the conserved nature of ubiquitination pathways across species, the use of HT29 cells provides additional insights into the dynamics of ubiquitination and miR-215 regulation during infection. To further investigate ubiquitination over time during the infection process, HT29 cells were included, with an additional time point at 24 h post-infection. In this model, ubiquitination was significantly inhibited by miR-215 mimic overexpression at both 2 h (*p* < 0.001) and 24 h (*p* < 0.05) post-infection, compared to infected mock controls (Figure 2C). These findings highlight the relevance of miR-215 in regulating ubiquitination in the context of *S*. Typhimurium infection and demonstrate the utility of complementary human cell models to validate and expand observations made in the porcine system.

**Figure 3:**
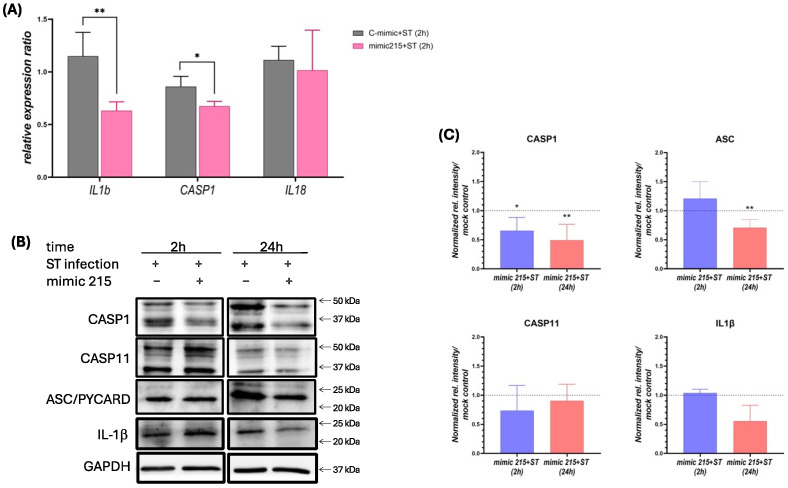
(**A**) Relative gene expression (to cyclophilin A) of inflammasome markers *IL1β*, *CASP1* and *IL18* in IPEC-J2 cells after mock or miR-215 mimic transfection and 2 h infection with *S*. Typhimurium. (**B**) Western blot of CASP1, ASC, CASP11 and IL1b in HT-29 cells transfected either with miR-215 or mock controls, 2h and 24h after infection by *S*. Typhimurium and (**C**) quantification of Western blot by expression ratio (ratio of relative intensity normalized to GAPDH compared to mock controls). Expression in transfected and infected samples were compared to their infected mock controls for each time point. * *p* < 0.05; **: *p* < 0.01.

Given the crucial role of ubiquitination in inflammasome activation, inflammasome markers were further analyzed. In IPEC-J2 cells, miR-215 overexpression during infection significantly reduced the *IL1β* and *CASP1* gene expression (*p* < 0.01 and *p* < 0.05, respectively) (Figure 3A). This reduction was confirmed at the protein level in HT29 cells, with a significant decrease in IL1β and CASP1 expression observed 2 h post-infection (*p* < 0.05) and further downregulation at 24 h post-infection (*p* < 0.01) (Figure 3B,C). Additionally, ASC was downregulated at 24 h (*p* < 0.01), while no significant changes were observed in CASP11 or IL1β at this later time point (Figure 3B,C).

**Figure 4:**
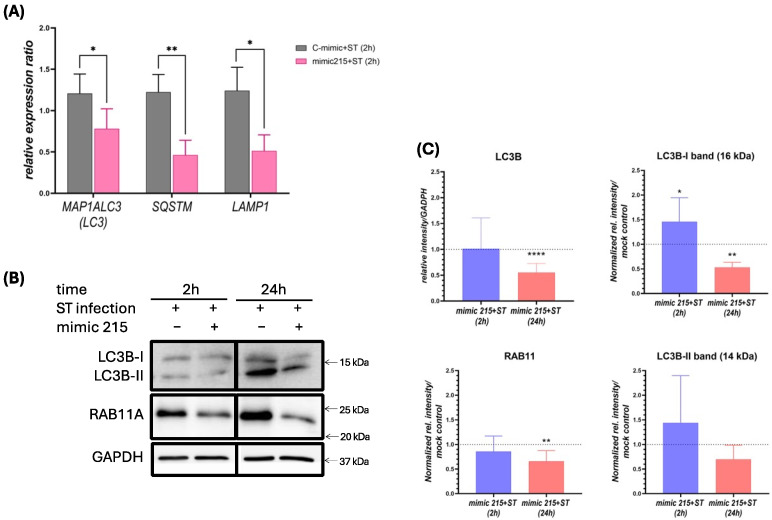
(**A**) Relative gene expression (to cyclophilin A) of autophagy markers *MAP1ALC3* (LC3), *SQSTM1* (p62) and *LAMP1* in IPEC-J2 cells after mock or miR-215 mimic transfection and 2 h infection with *S*. Typhimurium. (**B**) Western blot of LC3B and RAB11A in HT-29 cells transfected either with miR-215 or mock controls, 2 h and 24 h after infection by *S*. Typhimurium. (**C**) Quantification of Western blot by expression ratio (ratio of relative intensity normalized to GAPDH compared to mock controls). Expression in transfected and infected samples were compared to their infected mock controls for each time point. * *p* < 0.05; **: *p* < 0.01; ****: *p* < 0.0001.

Ubiquitination also plays a pivotal role in autophagy, as ubiquitinated proteins and pathogens are recognized and directed to autophagosomes for elimination. In IPEC-J2 cells, miR-215 mimic transfection during infection resulted in a significant reduction in genes coding for autophagy-related markers, including LC3B, SQSTM1 (sequestosome-1, p62) and lysosomal marker LAMP1 (Lysosomal-associated membrane protein 1) (Figure 4A). Consistently, in HT29 cells, protein expression of LC3B (specifically the LC3B-I form) and RAB11A also decreased significantly 24 h post-infection in miR-215 mimic-transfected cells (Figure 4B,C).

## 4. Discussion

Reducing the prevalence of *S*. Typhimurium in pigs is crucial for improving both animal and public health. However, the complexity of the interaction between *S*. Typhimurium and the host immune system, combined with the pathogen’s ability to evade host defenses, makes this a particularly challenging task. Our research on the role of miR-215 in regulating the innate immune response, especially in inflammasome activation and autophagy, provides new insights that could contribute to more effective control strategies.

Infection with *S*. Typhimurium leads to the differential expression of miRNAs, which regulate host response pathways. Some of the miRNAs identified in the present study (miR-215, miR-192, miR-9 and miR-31) have been previously reported to be altered during infection with a different *S*. Typhimurium isolate in pigs [11]. In our observations, we found similar expression patterns of certain miRNAs in both *S*. Typhimurium (ST) and mutant (MUT)-infected ileum, suggesting dysregulation in response to *Salmonella* infection. For instance, we found upregulation of miR-29b, which has been previously associated with positive regulation of *Shigella flexneri* infection, repressing UNC5C in a Rho-guanosine triphosphate hydrolases (GTPase)-dependent manner, thereby enhancing filopodia formation [39]. Similarly, upregulated miR-144 agrees with previous reports correlating its expression with inflammation in peripheral blood mononuclear cells from patients infected with *Mycobacteroides abscessus*, where it increased the expression of cytokines and chemokines such as IL1β, tumor necrosis factor (TNF), chemokine (C-X-C motif) ligand 2 (CXCL2) or interleukin 6 [40]. Also, miR-144-5p has been shown to modulate inflammatory response by targeting the NLR pathway, downregulating the NLRP3 inflammasome pathway [41].

Interestingly, some miRNAs were found differently expressed depending on the presence of *Salmonella* SPI2: miR-451a was overexpressed only in the SPI2 defective strain (MUT), while downregulation of the family of miR-215 (miR-215-5p, miR-215-3p and miR-192-3p) was found only in ST. As SPI2 is essential for intracellular survival and replication, we hypothesize that SPI2 may play a role in miRNA-mediated regulation during *Salmonella* infection. Contrary to our findings, a reduced expression of miR-451a has been previously found in COVID-19 patients, where miR451a was described as a down-regulator of the interleukin 6 receptor (IL6R), contributing to the cytokine storm characteristic of this disease [42]. Mir-451a has also been associated with other infections such as malaria [43].

Regarding miR-215, previous studies have reported a downregulation of this miRNA in porcine ileum during acute *S*. Typhimurium infection (log2FC = −4.1) [11]. Those results agree with the present study, where miRNA sequencing analysis of ST samples have revealed downregulation of all members of the miR-215/192 family, including miR-215-3p (FC = −2.9, *p* < 0.001), miR-215-5p (FC = −2.3, *p* < 0.05), miR-192-3p (FC = −2.9, *p* < 0.001) and miR-192-5p (FC = −1.6, *p* < 0.05). However, these findings contrast with a study on *Salmonella*-infected chicken ceca, in which the upregulation of miR-215-5p was reported [44]. Additionally, miR-215 has been found to be downregulated in skeletal muscle following lipopolysaccharide (LPS) challenge [45], and it has been suggested to regulate resistance to *Escherichia coli* in pigs [46]. MiR-215 is also associated with various biological processes and diseases, including pulmonary fibrosis [47] and several types of cancer [26,27]. Moreover, it has been identified as a regulator of inflammatory pathways, indicating that miR-215 may play a critical role in *Salmonella* infection [28,29,48]. However, the mechanism by which miR-215 is involved in the *Salmonella* infection process is still poorly understood. In related findings, it has been shown that hepatitis B virus can induce autophagy by downregulating miR-192-3p in hepatocellular carcinoma cell lines [49]. However, a study by Deng et al. observed the upregulation of miR-215 in *Mycobacterium tuberculosis* patients, concluding that this miRNA inhibits autophagosome-lysosome fusion in macrophages [29]. Interestingly, while only miR-215-5p had been reported in the porcine miRNA database, our work, by mapping the sequenced miRNAs to the human database, revealed the presence of miR-215-3p in porcine ileum, albeit at much lower expression than miR-215-5p.

To explore the function of miR-215-5p during *S*. Typhimurium infection, we used a mimic-based gain-of-function approach, identifying several potential targets involved in the ubiquitination process. Among these proteins we found UBE2I, also known as UBC9, which is a SUMO-conjugating enzyme, key in the SUMOylation pathway [50]. Depletion of UBC9 has been associated with overexpression of miR-30c and miR-30e during *S*. Typhimurium infection [51], suggesting that multiple miRNAs may regulate this protein. Another target, HUWE1, is an E3 ubiquitin ligase responsible for conjugating ubiquitin molecules to substrates [52], and we hypothesize that miR-215 may influence this process. Our ubiquitination assays indicated that miR-215 decreased ubiquitination both 2 h and 24 h after infection in porcine and human cell lines. Ubiquitination is a post translational modification (PTM) that can mark proteins for degradation or regulate processes such as endocytosis and intracellular trafficking [53]. This PTM can be modulated by the host or the pathogen during infection. *Salmonella* Typhimurium uses ubiquitination to counteract host immune responses [54], leading to selective autophagy, contributing to inflammasome assembly, or playing important roles in interferon- or NFκB-mediated immune signaling. The regulation of this process by miRNAs has been previously described. For instance, miR-339 targets ubiquitin-specific peptidase 8 (USP8), and has been reported to be involved in deubiquitination during tumorigenesis [55]. Similarly, let-7 downregulates E3 ubiquitin ligase TRIM71 in liver cancer [56]. Other miRNAs, including miR-7, miR-9 and miR-181c, are involved in the precise regulation of ubiquitination and deubiquitination processes in neurodegenerative diseases, where they interfere with proteasome-mediated degradation [57].

Ubiquitination plays a crucial role in the host autophagy process. It allows the host to ubiquitinate *Salmonella* for degradation, but *Salmonella* can also evade this process by manipulating or mimicking deubiquitinases, thereby reverting the PTM [54]. During *Salmonella* infection, the host cell attempts to remove unwanted cytosolic materials or bacteria through autophagy, forming autophagosomes that will fuse to lysosomes leading to degradation. In this selective process, ubiquitin binds to autophagy receptors (e.g., sequestosome-1 SQSTM1/p62 protein, optineurin) and microtubule-associated protein 1A/1B-light chain 3 (LC3), recruiting autophagy-related proteins (ATGs) and subsequently forming the autophagosome. During autophagy, the cytosolic form of LC3B (LC3B-I) is lipidated to form LC3B-II, which is associated with the autophagosomal membrane. LC3B-II is involved in the elongation and closure of the membrane, playing an essential role in autophagosome biogenesis. LC3B interacts with autophagy receptors that recognize and bind ubiquitinated cargo, ensuring the selective degradation of damaged organelles, misfolded proteins, and intracellular pathogens. We observed downregulation of LC3B-I but no changes in LC3B-II. We also found that miR-215 overexpression during infection leads to the decreased expression of the small GTPase RAB11, a small GTPase implicated in the maturation of autophagosomes by mediating autophagosomes–lysosome fusion, an essential step for the degradation of autophagic cargo [58]. RAB11 also facilitates the transport of immune receptors and other molecules to the cell surface, enhancing the cell’s ability to respond to pathogens [58].

Ubiquitination also regulates the assembly and activation of inflammasomes. Canonical NLRC4 and NLRP3 inflammasomes, as well as the non-canonical caspase-11 pathways, are activated in *S*. Typhimurium infection [18,59]. The inflammasome is a cytosolic multi-protein complex that induces inflammation and pyroptotic cell death in response to both pathogen (PAMPs) and endogenous activators (DAMPs). Recognition of PAMPs or DAMPs leads to formation of the inflammasome complex, which results in activation of caspase-1, followed by cleavage and release of pro-inflammatory cytokines such as IL-1β and IL-18. In this study, we found that miR-215 induces the downregulation of caspase-1 and ASC, suggesting a decrease in inflammasome activation through the canonical pathways, given that caspase-11 expression was not altered. Previous studies have shown that E3 ligases, such as TRIM31 and MARCH7, ubiquitinate NLRP3, targeting it for proteasomal degradation or modulating its activity [60,61]. The ubiquitination of ASC is crucial for the recruitment and activation of pro-caspase-1, leading to the processing and release of IL-1β and IL-18 [19]. Therefore, further studies on the ubiquitination of these proteins will provide additional information on this process.

The importance of this study in controlling *S*. Typhimurium infections in pigs lies in its potential to identify novel targets for intervention. Reducing the prevalence of *S*. Typhimurium in pigs is essential for improving animal health, food safety and public health, particularly as antibiotic resistance becomes a growing concern. By elucidating the role of miR-215 in regulating immune responses, such as autophagy and inflammasome activation, the research provides a deeper understanding of the molecular mechanisms involved in host–pathogen interactions. Additionally, by revealing how the pathogen manipulates host processes like ubiquitination and autophagy, the study offers insights into how *S*. Typhimurium evades immune responses, potentially opening the door for interventions that boost the host ability to clear the pathogen. This research holds promise for more effective control measures, reducing the burden of infection in pigs and ultimately mitigating the risk of zoonotic transmission to humans.

## 5. Conclusions

Enhanced understanding of how microRNAs regulate critical biological processes, such as ubiquitination, in response to infection paves the way for developing alternative therapies to combat this disease. In summary, this study found that miR-215 is down-regulated in *S*. Typhimurium infection, which may cause increased ubiquitination, autophagy and inflammasome activation during infection.

## Figures and Tables

**Table 1:** Top 10 differentially up- (red) and down-regulated (green) miRNAs in ileum samples from the two infected study groups (ST and MUT).

	ID	Fold Change	*p*-Value	FDR
ST/C	miR-29b-3p	2.83	1.6 × 10^−7^	4.9 × 10^−5^
	miR-144-5p	2.65	9.0 × 10^−5^	5.7 × 10^−3^
	miR-181b-3p	2.50	2.2 × 10^−4^	9.9 × 10^−3^
	miR-21-3p	2.36	6.9 × 10^−6^	1.1 × 10^−3^
	miR-301a-3p	2.14	4.6 × 10^−4^	1.6 × 10^−2^
	miR-144-3p	2.11	2.3 × 10^−3^	4.3 × 10^−2^
	miR-374b-3p	2.07	5.9 × 10^−4^	1.9 × 10^−2^
	miR-9-3p	2.07	1.1 × 10^−3^	2.8 × 10^−2^
	miR-301b-3p	2.05	3.3 × 10^−3^	4.7 × 10^−2^
	miR-20b-3p	1.99	7.3 × 10^−3^	7.5 × 10^−2^
	miR-31-3p	−2.00	2.0 × 10^−2^	1.3 × 10^−1^
	miR-582-5p	−2.18	1.7 × 10^−3^	3.8 × 10^−2^
	miR-31-5p	−2.20	4.2 × 10^−4^	1.6 × 10^−2^
	miR-215-5p	−2.34	1.6 × 10^−2^	1.2 × 10^−1^
	miR-676-3p	−2.53	4.3 × 10^−5^	3.4 × 10^−3^
	miR-574-5p	−2.54	1.8 × 10^−4^	9.4 × 10^−3^
	miR-196b-5p	−2.72	1.3 × 10^−1^	3.7 × 10^−1^
	miR-196a-5p	−2.78	6.2 × 10^−2^	2.4 × 10^−1^
	miR-215-3p	−2.87	2.3 × 10^−3^	4.3 × 10^−2^
	miR-192-3p	−2.93	1.5 × 10^−5^	1.6 × 10^−3^
MUT/C	miR-451a	2.44	3.6 × 10^−3^	5.9 × 10^−1^
	miR-184	1.97	2.4 × 10^−2^	9.4 × 10^−1^
	miR-144-5p	1.83	4.0 × 10^−2^	9.4 × 10^−1^
	miR-374b-3p	1.77	5.6 × 10^−3^	5.9 × 10^−1^
	miR-20b-3p	1.69	3.8 × 10^−2^	9.4 × 10^−1^
	miR-144-3p	1.59	8.9 × 10^−2^	9.7 × 10^−1^
	miR-301b-3p	1.57	6.5 × 10^−2^	9.4 × 10^−1^
	miR-181b-3p	1.56	5.6 × 10^−2^	9.4 × 10^−1^
	miR-486-5p	1.54	2.2 × 10^−2^	9.4 × 10^−1^
	miR-29b-3p	1.52	2.4 × 10^−2^	9.4 × 10^−1^
	miR-676-3p	−1.91	5.6 × 10^−3^	5.9 × 10^−1^
	miR-802	−1.88	1.8 × 10^−2^	9.4 × 10^−1^
	miR-141-3p	−1.70	5.9 × 10^−2^	9.4 × 10^−1^
	miR-31-5p	−1.61	6.9 × 10^−2^	9.4 × 10^−1^
	miR-22-3p	−1.58	2.8 × 10^−2^	9.4 × 10^−1^
	miR-615-3p	−1.57	6.5 × 10^−2^	9.4 × 10^−1^
	miR-574-5p	−1.56	5.6 × 10^−2^	9.4 × 10^−1^
	miR-200c-5p	−1.55	9.2 × 10^−2^	9.7 × 10^−1^
	miR-200c-3p	−1.55	3.1 × 10^−2^	9.4 × 10^−1^
	miR-10b-3p	−1.49	4.0 × 10^−2^	9.4 × 10^−1^

## Data Availability

Sequencing data have been deposited at National Institutes of Health Sequencing Reading Archive (NCBI SRA) database under Bioproject accession ID PRJNA1160452. The mass spectrometry proteomics data have been deposited to the ProteomeXchange Consortium via the Proteomics Identification Database (PRIDE) (81) partner repository with the dataset identifier PXD054446.

## Supplementary Materials

The following supporting information can be downloaded at: https://www.mdpi.com/article/10.3390/ani15030431/s1. Table S1: Reads and differential expression analysis from small RNA-seq of ST- and MUT-infected ileum. Table S2: Results from LFQ proteomics of miR-215 mimic transfected and ST-infected IPEC-J2 cells. Table S3: Primers used in this study. Figure S1: (A) Sequencing reads from porcine ileum mapped to human miRNA database. (B) alignment of human and porcine mature miR-215-3p sequence. (C) Structures of human and porcine stem-loop sequences, with the only human-porcine sequence mismatch highlighted.
