# Supplementary material for: miR-215 Modulates Ubiquitination to Impair Inflammasome Activation and Autophagy During Salmonella Typhimurium Infection in Porcine Intestinal Cells
Source: Animals (Basel). 2025 Feb 4;15(3):431. doi: 10.3390/ani15030431 (PMC11815736; doi:10.3390/ani15030431)
Supplement: Supplementary file 1 [file animals-15-00431-s001.zip › animals-3407027-supplementary/Figure S1.pptx]

## Slide 1
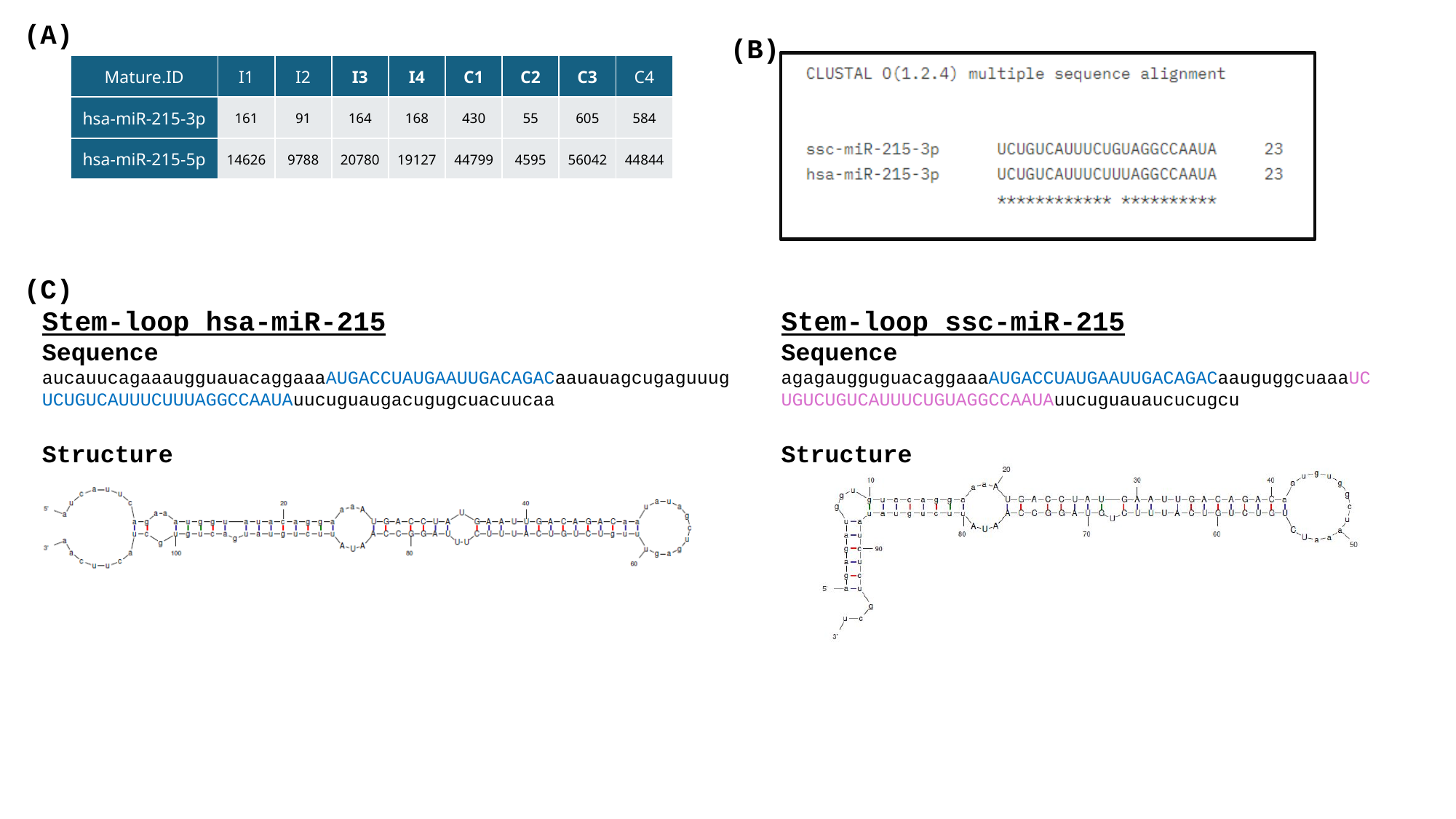

(A)
(B)
| Mature.ID | I1 | I2 | I3 | I4 | C1 | C2 | C3 | C4 |
| --- | --- | --- | --- | --- | --- | --- | --- | --- |
| hsa-miR-215-3p | 161 | 91 | 164 | 168 | 430 | 55 | 605 | 584 |
| hsa-miR-215-5p | 14626 | 9788 | 20780 | 19127 | 44799 | 4595 | 56042 | 44844 |
(C)
Stem-loop ssc-miR-215
Sequence
agagaugguguacaggaaaAUGACCUAUGAAUUGACAGACaauguggcuaaaucugucugucauuucuguaggccaauauucuguauaucucugcu
Structure
Stem-loop hsa-miR-215
Sequence
aucauucagaaaugguauacaggaaaAUGACCUAUGAAUUGACAGACaauauagcugaguuugUCUGUCAUUUCUUUAGGCCAAUAuucuguaugacugugcuacuucaa
Structure
